# Differential expression profiles of miRNAs and their putative targets in *Schistosoma mansoni* during its life cycle

**DOI:** 10.1590/0074-02760200326

**Published:** 2021-05-14

**Authors:** Fabiano CP Abreu, Ester Alves Mota, Roberta V Pereira, Victor F Oliveira, Marcela P Costa, Matheus de S Gomes, Liana K Jannotti-Passos, William C Borges, Renata Guerra-Sá

**Affiliations:** 1 Universidade Federal de Ouro Preto, Núcleo de Pesquisas em Ciências Biológicas, Ouro Preto, MG, Brasil; 2 Universidade Federal de Uberlândia, Instituto de Genética e Bioquímica, Patos de Minas, MG, Brasil; 3 Fundação Oswaldo Cruz-Fiocruz, Centro de Pesquisas René Rachou, Belo Horizonte, MG, Brasil

**Keywords:** miRNA, differential expression, Schistosoma mansoni, miRNA target genes

## Abstract

**BACKGROUND:**

Schistosomiasis is a disease caused by *Schistosoma*. Due to its complex life cycle, evolutionary position and sexual dimorphism, schistosomes have several mechanisms of gene regulation. MicroRNAs (miRNAs) are short endogenous RNAs that regulate gene expression at the post-transcriptional level by targeting mRNA transcripts.

**OBJECTIVES:**

Here, we tested 12 miRNAs and identified their putative targets using a computational approach.

**METHODS:**

We performed the expression profiles of a set of miRNAs and their putative targets during the parasite’s life cycle by quantitative reverse transcription-polymerase chain reaction (qRT-PCR).

**FINDINGS:**

Our results showed differential expression patterns of the mature miRNAs *sma-miR-250*; *sma-miR-92a*; *sma-miR-new_4-3p*; *sma-miR-new_4-5p*; *sma-miR-new_5-5p*; *sma-miR-new_12-5p*; *sma-miR-new_13-3p* and *sma-miR-new_13-5p*. Interestingly, many of the putative target genes are linked to oxidative phosphorylation and are up-regulated in adult-worms, which led us to suggest that miRNAs might play important roles in the post-transcriptional regulation of genes related to energetic metabolism inversion during parasite development. It is noteworthy that the expression of *sma-miR-new_13-3p* exhibited a negative correlation on *SmNADH:ubiquinone oxidoreductase complex I*.

**MAIN CONCLUSIONS:**

Our analysis revealed putative miRNA genes related to important biological processes, such as transforming growth factor beta (TGF-β) signaling, proteasome regulation, glucose and lipid metabolism, immune system evasion and transcriptional regulation.

Schistosomiasis is a chronic debilitating disease that is caused by blood flukes of the genus *Schistosoma* and affects more than 200 million individuals in tropical and subtropical regions. The complex life-cycle of schistosomes, which includes sexual and asexual reproduction within vertebrate and invertebrate hosts, is a difficult challenge for the control of this parasitic disease.^1^ The parasite has seven distinct developmental stages, four within the definitive host (schistosomula, adult male and female worms and eggs) and one within the intermediate host (sporocysts), as well the free-living forms cercariae and miracidium, which are the infective forms of the vertebrate and the invertebrate hosts, respectively. To complete the parasitic life-cycle, mechanisms of gene expression control are extremely important, particularly transcriptional and post-transcriptional regulation.^2^ ^,^ ^3^ One of the important mechanisms of post-transcriptional gene regulation is the degradation or translational inhibition of mRNAs by microRNAs (miRNAs).^3^

MiRNAs are a class of small non-coding RNAs (18-25 nucleotides) that post-transcriptionally regulate gene expression by base-pairing to the 3’untranslated region (3’UTR) of target messenger RNAs. The binding of miRNA to a target mRNA leads to translation repression and/or exonucleolytic mRNA decay.^4^ The importance of miRNAs for development is highlighted by the fact that miRNAs consist of approximately 1% of animals’ genes and are highly conserved across a wide range of species.^4^ MiRNAs have been identified in the genus *Schistosoma*, which includes *S. japonicum*, *S. haematobium* and *S. mansoni*.^5^ ^,^ ^6^ ^,^ ^7^ ^,^ ^8^ ^,^ ^9^ ^,^ ^10^ Several groups have identified and described miRNAs in *S. japonicum* using the deep sequencing technique that generates a great amount of miRNA data.^9^ ^,^ ^10^ Studies suggested that Schistosome miRNAs play a potentially regulatory role in worm development, parasitism and in drug resistance.^11^

Our previous studies have provided a systematic analysis of miRNAs and have identified 67 mature and 42 miRNA precursors in *S. mansoni* using an integrated computational approach.^6^ A second recent report identified 211 novel miRNAs in *S. mansoni* by cloning small RNA sequences from adults and schistosomula.^8^ Marco et al.^12^ who used deep sequencing, identified 112 miRNAs in adult worms of *S. mansoni* and confirmed 20 of the 42 precursor miRNAs that were identified by our group and two of the 211 that were reported by Simões et al.^8^ The miRNA targets and expression profile analyses in males and females suggested that one of the sex-specific miRNAs (sma-miR-71) might be involved in female sex-specific functions.^6^ ^,^ ^12^ Furthermore, our group showed by computational approaches that the miR-71/2 cluster is highly conserved across the Protostomia phyla and verified that the miR-2 family is completely absent from Deuterostomia species, whereas the miR-71 family is absent from Vertebrata and Urochordata.^6^ These results were corroborated by Marco et al.,^12^ who showed the origin of miRNAs of *S. mansoni*, demonstrating that miR-71 and miR-2 families are protostome specific. Continuing this investigation, here we aimed to evaluate the expression of twelve miRNAs previously identified by our group (four conserved and eight non-conserved) in cercariae, early schistosomula and adult worms. Additionally, we also identified the biologically relevant targets of the miRNAs in order to understand the functions of these regulatory molecules. Our results showed differential expression patterns of the miRNAs *sma-miR-250*; *sma-miR-92a*; *sma-miR-new_4-3p*; *sma-miR-new_4-5p*; *sma-miR-new_5-5p*; *sma-miR-new_12-5p*; *sma-miR-new_13-3p* and *sma-miR-new_13-5p*.

## MATERIALS AND METHODS

*Parasites* - The *S. mansoni* logarithmic strain (LE) was maintained by routine passage through *Biomphalaria glabrata* snails and BALB/c mice. Infected snails were induced to shed cercariae under light exposure for 2 h, which was followed by the recovery of the larvae by sedimentation on ice. Adult worm parasites were obtained by liver perfusion of mice after infection for 50 days.^13^ Mechanically transformed schistosomula (MTS) were prepared as previously described by Harrop and Wilson.^14^ Briefly, cercariae were recovered, washed in RPMI-1640 medium (Invitrogen, São Paulo, Brazil) and then vortexed at maximum speed for 90 s. The cercariae were immediately cultured for 3.5 h at 37ºC with 5% CO_2_. The recovered schistosomula were washed with RPMI-1640 until no tails were detected. For the subsequent incubations, the parasites were maintained in M169 medium, which was supplemented with 10% fetal bovine serum, penicillin (100 μg/mL), streptomycin (100 μg/mL) and 5% Schneider’s medium^15^ at 37ºC with 5% CO_2_ for 3.5 and 24 hours.

*Analysis of miRNA expression* - The total RNA samples that were enriched with miRNAs from cercariae, schistosomula and adult worms were obtained using a miRNeasy Mini Kit (Qiagen). The preparation was treated with RNase-free DNase I. RNA was quantified using a spectrophotometer and the cDNAs were obtained using 1 µg of total RNA by reverse transcription using a miScript II RT Kit (Qiagen).

Reverse-transcribed cDNA samples were used as templates for polymerase chain reaction (PCR) amplification using a miScript SYBR Green PCR Kit (Qiagen) and a 7300 Real Time PCR System (Applied Biosystems). The mature miRNA sequences were used as primers and the non-coding U6 gene sequence was used as the endogenous control. The mature miRNA sequences are provided in the Supplementary data (Tables I-II). For the investigated transcripts, three biological replicates were performed and their expression levels were relative to the U6^16^ transcript according to the 2^−ΔCt^ method^17^ using the Applied Biosystems 7300 software.

*Computational analysis and prediction of miRNA target genes* - Sequences of mature miRNA genes (*sma-miR-281*; *sma-miR-250*, *sma-miR-2162-3p*; *sma-miR-92a*; *sma-miR-new_2-5p*; *sma-miR-new_4-3p*; *sma-miR-new_4-5p*; *sma-miR-new_5-5p*; *sma-miR-new_12-5p*; *sma-miR-new_13-3p*; *sma-miR-new_13-5p* and *sma-miR-new_16-3p*) were searched through the miRBase database version 20.0 (http://www.mirbase.org/) using the cut-off ≥ 0.05 to evaluate the miRNA conservation among other organisms.^18^ To obtain the miRNA target genes, the 3’UTR-sequences were collected from the database GeneDB (http://www.genedb.org/genedb/smansoni/) and the miRanda software version 3.3a, which was released in 2010, was used to search for targets within the following parameters and conditions: a gap opening penalty of -8; a gap extension penalty of -2; a match minimum score threshold of 120; a target duplex with a maximum threshold free energy of -15 Kcal/mol; a scaling parameter of 3 for the complementary nucleotide match score; counting from the miRNA 5’end; and demanding a strict 5’ seed pairing between two and eight nucleotides.

The predicted target genes were retrieved from the *S. mansoni* database version 5.0 from GeneDB and the ontologies were obtained from the Gene Ontology database (http://www.geneontology.org/). Moreover, Kyoto encyclopedia of genes and genomes (KEEG) was used to include functional information regarding the target genes. The software TargetScan, version 6.2, which was released in 2012 (http://www.targetscan.org/), was used to obtain *Homo sapiens* and *Mus musculus* miRNAs that are involved in identical biological functions as in *S. mansoni*.

*Expression analysis of miRNA target genes* - Total RNA from cercariae, schistosomula and adult worms was obtained using a combination of the Trizol reagent (Sigma) and chloroform for extraction and the RNA was column-purified using the SV total RNA Isolation system (Promega). The preparation was treated with RNase-free DNase I (RQ1 DNase; Promega). The RNA was quantified using a spectrophotometer and an aliquot containing 1 *μg* of total RNA was reverse transcribed using an oligo dT primer from the Thermoscript reverse transcription-polymerase chain reaction (RT-PCR) System (Invitrogen), as described by the manufacturer. The efficiency of DNAse I treatment was evaluated by PCR amplification of the cDNA reaction mix without the addition of the ThermoScript enzyme. *S. mansoni*-specific primers were designed using the program GeneRunner^*®*^ . The sequence accessions and primer pairs are shown in Supplementary data (Tables I-II). RT-cDNA samples were used as the templates for PCR amplification with the SYBR Green Master Mix UDG-ROX^*®*^ (Invitrogen) and 7300 Real-time PCR System (Applied Biosystems). Specific primers for *S. mansoni* EIF4E were used as an endogenous control (GeneDB ID: Smp_001500) (forward, 5’TGTTCCAACCACGGTCTCG3’; reverse, 5’TCGCCTTCCAATGCTTAGG3’).^19^ The efficiency of each pair of primers was evaluated according to the protocol developed by Applied Biosystems (cDNA dilutions 1:4, 1:16, 1:64, 1:256, and 1:1024). For all investigated transcripts, three biological replicates were performed and gene expression was relative to the EIF4E transcript according to the 2^-ΔCt^ method^17^ using Applied Biosystems 7300 software [Supplementary data (Table III)].

*Statistical analyses* - The statistical analyses were performed using the GraphPad Prism version 5.0 software package (Irvine, CA, USA). The normality of the data was established using a one-way analysis of variance (ANOVA). Tukey’s post hoc tests were used to investigate if the differential expressions of transcripts throughout the investigated stages were significant. The differences were considered significant when p values were < 0.05. The correlation between expression of targets and miRNAs was made using Spearman considering a significance when p values were < 0.05.

*Ethics statement* - All experiments involving animals were permitted by the Ethical Committee for Animal Care of the Federal University of Ouro Preto (protocol 2011/55). These procedures were conducted in accordance with the accepted national and international regulations for laboratory animal use and care (Law nº 11.794, 10/08/2008).

## RESULTS

**Fig. 1:**
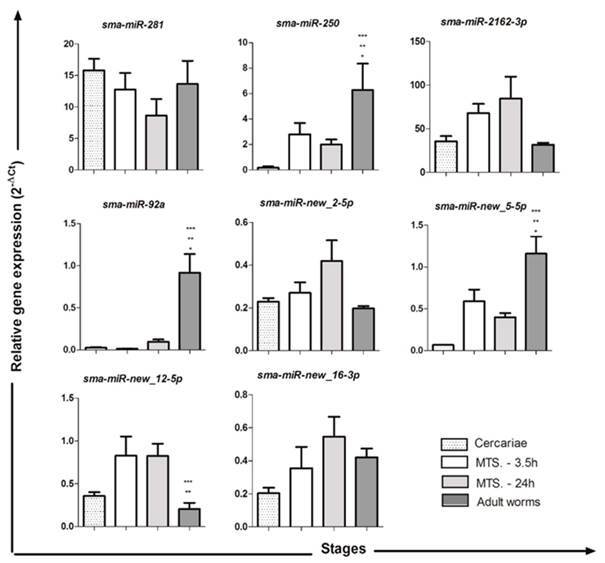
miRNA expression profiles throughout the *Schistosoma mansoni* life cycle. The miRNA expression levels were measured based on three replicates during the following stages: cercariae, MTS-3.5 h, MTS-24 h and adult worms using quantitative reverse transcription polymerase chain reaction (qRT-PCR). Expression levels were calibrated according to the comparative 2^−ΔCt^ method and used the constitutively expressed non-coding U6 gene as an endogenous control (one-way variance analysis followed by Tukey’s pairwise comparison, p < 0.05).

**Fig. 2:**
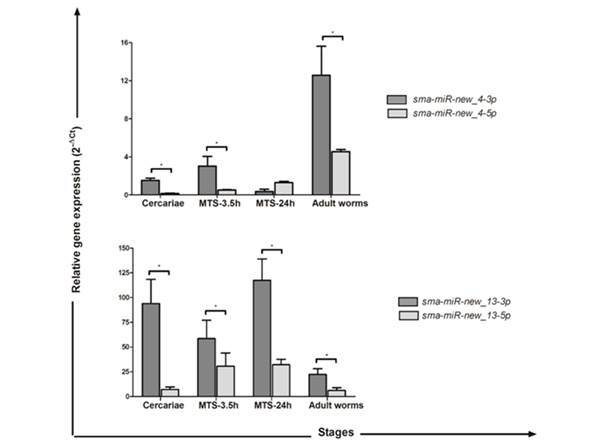
*Sma-miR-new_4-3p*, *sma-miR-new_4-5p*, *sma-miR-new_13-3p* and *sma-miR-new_13-5p* expression profiles throughout the *Schistosoma mansoni* life cycle. The miRNA expression levels were measured based on three replicates during the following stages: cercariae, MTS-3.5 h, MTS-24 h and adult worms using quantitative reverse transcription polymerase chain reaction (qRT-PCR). Expression levels were calibrated according to the comparative 2^−ΔCt^ method and used the constitutively expressed non-coding U6 gene as an endogenous control (two-way variance analysis, p < 0.0001); *significant difference.

*Expression profile of miRNAs in different S. mansoni stages* - The gene expression of twelve miRNAs (*sma-miR-281*; *sma-miR-250*, *sma-miR-2162-3p*; *sma-miR-92a*; *sma-miR-new_2-5p*; *sma-miR-new_4-3p*; *sma-miR-new_4-5p*; *sma-miR-new_5-5p*; *sma-miR-new_12-5p*; *sma-miR-new_13-3p*; *sma-miR-new_13-5p* and *sma-miR-new_16-3p*) was evaluated using quantitative reverse transcription-polymerase chain reaction (qRT-PCR) with three technical and three biological replicates. It is important to highlight that those miRNAs presenting “new” in their nomenclature are not conserved among other species and probably are new specific miRNAs of the *Schistosoma* genus. Previously, our group described 63 mature and 42 precursor miRNAs in *S. mansoni* and showed the expression profiles of eight miRNAs. MiRNAs play a distinct role in modulating the growth, sexual maturation and reproduction.^10^ ^,^ ^13^ The detection of their putative targets is the most common and the first step to understand their roles. Here, we aimed to evaluate the expression profile of twelve mature miRNAs that have been not tested yet, among those 63 that were predicted by our group. Only nine of them are expressed both in larval phases and adult worms.^6^ The results showed differential miRNA expression profiles among the stages, except for *sma-miR-281*, *sma-miR-2162-3p*, *sma-miR-new_2-5p* and *sma-miR-new_16-3p*, which showed no significant differences (Figs 1-2). We observed higher transcriptional levels of *sma-miR-250*, *sma-miR-92a* and *sma-miR-new_5-5p* in adult worms compared to other stages, whereas *sma-miR-new_12-5p* was primarily expressed in schistosomula stage (MTS-3.5 h and 24 h) compared with cercariae and adult worms. Furthermore, we observed that both arms of *sma-miR-new_4* and *sma-miR-new_13* were expressed among the stages evaluated (Fig. 2). Both sma-*miR-new_4-3p* and *sma-miR-new_4-5p* presented higher transcriptional levels in adult worms compared to other stages. *Sma-miR-new_13-3p* showed increased expression profiles in larval stages and a down-regulation in adult worms, while *sma-miR-new_13-5p* was up-regulated in schistosomula stage (MTS-3.5 h and 24 h) compared to cercariae and adult worms. Further, the *sma-miR-new_13-3p* showed transcriptional levels up to 100-fold higher than *sma-miR-new_13-5p* among the stages. Finally, the *sma-miR-2162-3p* and *sma-miR-new-13-3p* are the most expressed of the twelve mature miRNAs investigated in this work.

*Prediction of miRNA target genes* - The mature miRNA sequences were basic local alignment search tool (BLAST) searched through the miRBase database and, except for *sma-miR-250*, *sma-miR-281*, *sma-miR-92a* and *sma-miR-2162-3p*, all other miRNAs showed no conservation among other species. The miRNA target genes were predicted using the miRanda software and the results were filtered according to the parameters that were established. We obtained a list of target genes for each miRNA evaluated and selected those ones that are thought be important in *S. mansoni* biology. Further, we verified the ontology based on molecular functions and biological processes using Gene Ontology and KEGG tools (Table). The prediction showed relevant target genes for seven of the twelve miRNAs evaluated here (Table). The miRNA target genes prediction revealed genes related to important biologic processes as energetic and lipid metabolism, proteasome regulation, immune system evasion and transcriptional regulation in the parasite. Interestingly, we found several miRNA target genes involved in the oxidative phosphorylation process in mitochondria like miRNAs targets related to the oxidative phosphorylation process in the respiratory chain. Additionally, we observed some targets that are involved in other important pathways in the parasite, which include the transforming growth factor beta (TGF-β) pathway, proteasome inhibition (PI31), lipid metabolism, glucose metabolism, a surface protein (VAL) and a digestive host blood protein (Cathepsin).

**TABLE:** Association between miRNAs, their putative targets, locations and molecular functions

miRNA ID	Target	GeneDB	Biological process	Molecular function	Celular component	Ortholog function
sma-mir-281	TGF- β signal transducer smad2	Smp_085910	TGF-β pathway (GO:0007179)	Sequence-specific DNA binding transcription factor activity (GO:0003700)	Cytoplasm (GO:0005737)	hsa-miR-425 (NM_001003652)
sma-mir-new_2-5p	ATP synthase lipid-binding protein-like protein	Smp_000880.1/2	Oxidative Phosphorylation (GO:0006119)	Proton-transporting ATP synthase activity (GO:0046933)	Mitochondrion (GO:0005739)	hsa-miR-3686 (NM_001001937)
	Ubiquinone biosynthesis protein	Smp_001430	Ubiquinone biosynthetic process (GO:0006744)	Protein kinase activity (GO:0004672)	Mitochondrion (GO:0005739)	hsa-miR-548m/hsa-let-7a (NM_016035)
	*Lipase maturation* *factor*	Smp_026910.1/2	Protein maturation (GO:0051604)	-----	Endoplasmic reticulum (GO:0005783)	hsa-miR-124/mmu-miR-124 (NM_033200)
	*NADH* *dehydrogenase (ubiquinone)* *Fe-S protein 7*	Smp_092490	Oxidative Phosphorylation (GO:0006119)	NADH dehydrogenase (ubiquinone) activity (GO:0008137)	Mitochondrion (GO:0005739)	hsa-mir-548e/mmu-miR-871-5p (NM_001199981)
	*NADH dehydrogenase (ubiquinone) 1 beta*	Smp_036400.1	Oxidative Phosphorylation (GO:0006119)	NADH dehydrogenase (ubiquinone) activity (GO:0008137)	Mitochondrion (GO:0005739)	mmu-miR-541 (NM_005004)
sma-mir-new_4-5p	Cytochrome c type heme lyase	Smp_022320.1	Oxidative Phosphorylation (GO:0006119)	Holocytochrome-c synthase activity (GO:0004408)	Mitochondrion (GO:0005739)	hsa-miR-429/mmu-miR-200c (NM_001122608)
	*Proteasome inhibitor PI31 subunit*	Smp_066340.1	Regulation of proteasome assembly (GO:0090364)	Endopeptidase inhibitor activity (GO:0004866)	Proteasome core complex (GO:0005839)	hsa-miR-181a/mmu-miR-181b (NM_006814)
sma-mir-new_5-5p	Lipoic acid synthetase	Smp_010100	Protein lipoylation (GO:0009249)	Lipoate synthase activity (GO:0016992)	Mitochondrion (GO:0005739)	hsa-miR-493(NM_194451)
sma-mir-new_12-5p	*Tetraspanin*, *putative*	Smp_174630	-----	-----	Integral to membrane (GO:0016021)	hsa-miR-3189-3p/mmu-miR-3058 (NM_147161)
	*Acyl coenzyme A thioesterase*	Smp_213500	Acyl-CoA metabolic process (GO:0006637)	Acyl-CoA thioesterase activity (GO:0047617)	Peroxisome (GO:0005777)	------
	*Mitochondrial carrier 2*	Smp_053800	Transport (GO:0006810)	Transporter activity (GO:0005215)	Mitochondrion (GO:0005739)	hsa-miR-150/mmu-miR-5127 (NM_014342)
	*Cathepsin B peptidase 1*	Smp_085010	Proteolysis (GO:0006508)	Cysteine-type peptidase activity (GO:0008234)	Lysosome (GO:0005764)	hsa-miR-7/mmu-miR-761 (NM_001908)
sma-mir-new_13-3p	NADH:ubiquinone oxidoreductase complex I	Smp_170410	Oxidative Phosphorylation (GO:0006119)	NADH dehydrogenase (ubiquinone) activity	Mitochondrion (GO:0005739)	hsa-miR-4477a (NM_004541)
	*Insulin growth factor binding protein 7*	Smp_180600	Regulation of cell growth (GO:0001558)	Insulin-like growth factor binding (GO:0005520)	Extracellular region (GO:0005576)	hsa-miR-3935/mmu-miR-688 (NM_001553)
sma-mir-new_13-5p	Venom allergen-like (VAL) 6 protein	Smp_124050.1/2/3	Pathogenesis (GO:0009405)	Ion channel inhibitor activity (Go:0008200)	Extracellular region (GO:0005576)	-----

**Fig. 3:**
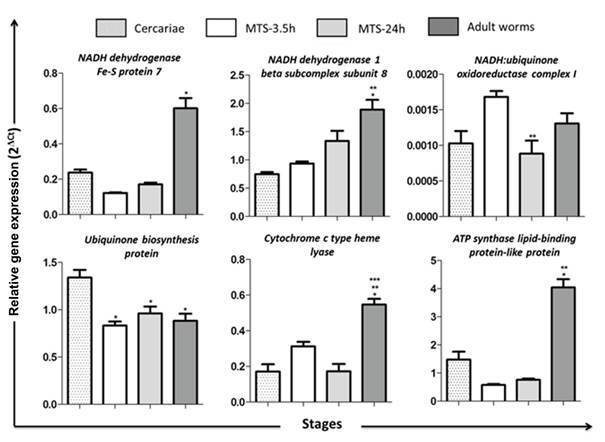
miRNA target genes expression profiles throughout the *Schistosoma mansoni* life cycle. Expression levels were measured and using quantitative reverse transcription polymerase chain reaction (qRT-PCR) calibrated according to the comparative 2^−ΔCt^ method using the constitutively expressed EIF4E gene as an endogenous control based on three replicates during the stages: cercariae, MTS-3.5 h, MTS-24 h and adult worms. Targets of *sma-miR-new_2-5p*: *SmATP synthase lipid-binding protein-like protein*, *Smubiquinone biosynthesis protein*, *SmNADH dehydrogenase Fe-S protein 7*, *SmNADH dehydrogenase 1 beta subcomplex subunit 8*; target of *sma-miR-new_4-5p*: *Smcytochrome c type heme lyase* and the target of *sma-miR-new_13-3p*: *SmNADH:ubiquinone oxidoreductase complex I*. *Different from cercariae; **different from MTS-3.5 h; ***different from MTS-24 h (one-way variance analysis followed by Tukey’s pairwise comparison, p < 0.05).

**Fig. 4:**
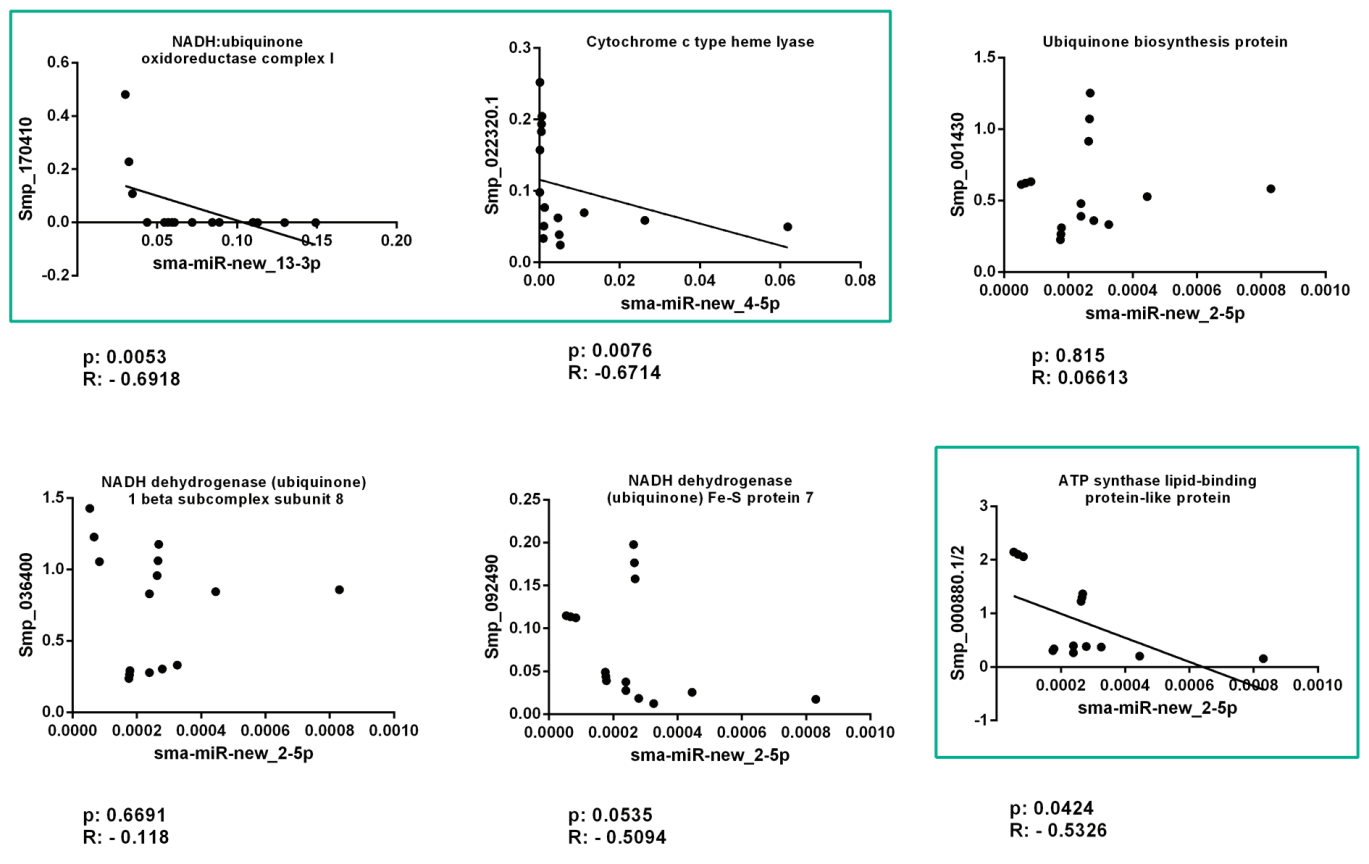
Spearman correlation test between the miRNA expression and the expression of their respective putative targets. The expression levels of the targets were measured and using quantitative reverse transcription polymerase chain reaction (qRT-PCR) calibrated according to the comparative 2^−ΔCt^ method using the constitutively expressed EIF4E gene as an endogenous control based on three replicates during the stages and for the miRNAs the endogenous control used was snU6. The analysis was conducted with p < 0.05, the boxes show the significant negative correlations.

*Differential expression of miRNA target genes* - We accessed the expression profiles of six mRNAs: *SmNADH dehydrogenase Fe-S protein 7*, *SmNADH dehydrogenase 1 beta subcomplex subunit 8*, *SmATP synthase lipid-binding protein-like protein*, *Smubiquinone biosynthesis protein* are potential targets of *sma-miR-new_2-5p*; *Smcytochrome c type heme lyase* were identified as a target of *sma-miR-new_4-5p*, and *SmNADH:ubiquinone oxidoreductase complex I* was identified as a target of *sma-miR-new_13-3p*. The results were expressed using mRNA levels in relation to EIF4E gene as the endogenous control (Fig. 3). The results showed differential mRNA expression profiles among the stages. We observed a higher expression profile of *SmNADH dehydrogenase Fe-S protein 7*, *SmNADH dehydrogenase 1 beta subcomplex subunit 8*, *Smcytochrome c type heme lyase* and *SmATP synthase lipid-binding protein-like* protein in adult worms compared with all other stages. *SmNADH:ubiquinone oxidoreductase complex I* presented a significant lower expression in schistossomula 24 h. Furthermore, Smubiquinone biosynthesis protein showed up-regulation in cercariae stage compared with schistosomula and adult worms. We found three negative correlations between miRNAs and their targets, using Spearman test, p < 0.05: *NADH:ubiquinone oxidoreductase complex I* (Smp_170410) correlated with *sma-miR-new_13-3p*; *cytochrome c type heme lyase* (Smp_022320.1) correlated with *sma-miR-new_4-5p* and *ATP synthase lipid-binding protein-like* (Smp_000880.1/2) correlated with *sma-miR-new_2-5p* (Fig. 4).

## DISCUSSION

In this work, we described the expression profiles of twelve *S. mansoni* miRNAs by measuring their transcript levels using qRT-PCR during the developmental stages of cercariae, schistosomula (3.5 and 24 h) and adult worms. The results showed differential miRNA transcript levels among the stages for eight mature miRNAs. The *sma-miR-2162-3p* and *sma-miR-new_13-3p* were the most highly expressed miRNAs compared to the others. Interestingly, both arms of *sma-miR-new_4* (*sma-miR-new_4-3p* and *sma-miR-new_4-5p*) and *sma-miR-new_13* (*sma-miR-new_13-3p* and *sma-miR-new_13-5p*) showed significant transcriptional levels and are differentially expressed, which suggests that both arms might be functional. The selection of a mature miRNA arm (3p or 5p) may vary between different tissues, development stages and species.^3^ Recently, the sex-biased expression pattern of certain miRNAs, as shown by qRT-PCR validation in *S. mansoni*, indicated that *sma-miR-281* is male-specific.^12^ In *Bombyx mori*, an insect, the miR-281 seems to be involved in regulation of the ecdysone receptor (EcR) isoform B during the larval molt and metamorphosis in the Malpighian tubules.^20^ Marco et al.^12^ also showed by molecular phylogeny that the *sma-sma-miR-281* and *miR-2162* are specific to protostomes and platyhelminthes, respectively. Furthermore, strongly biased expression patterns of *sja*-miR-71 and *sja*-miR-36 family members in eggs suggest roles of these small RNAs in embryonic development.^21^ Marco et al.^12^ also demonstrated sex-biased expression profiles of the miR-71 and miR-36 families in females. These data, together with our analysis, reinforce the hypothesis that miRNAs play a distinct role in modulating the development, maturation and reproduction of this parasite. In addition, we used the freely available prediction program miRanda to predict the miRNAs target genes and categorised them accordingly to the gene ontology.

Since the 1970s, it has been observed that *S. mansoni* changes its metabolism by alternating between the use of stored glycogen and the reliance on host glucose to supply its energy requirements.^22^ It is generally accepted that the free-living stages, such as eggs, miracidium and cercariae, are primarily dependent on the Krebs cycle and respiratory chain activity for their energy generation.^22^ Studies suggest that the biological transformation of cercariae to schistosomula is accompanied by a transition from aerobic to a more anaerobic energy metabolism.^23^ The schistosomula produce mainly lactate and pyruvate as the products of the degradation of external glucose.^23^ The increased formation of lactate is induced by the presence of external glucose, which results in an increased glycolytic flux.^24^ In the vertebrate blood stream, the schistosomula develop into adult worms, which generate much lactate. The parasite remains a homo-lactate fermentor for the rest of its life inside the final host.^24^ In this work, four of the twelve miRNAs evaluated here (*sma-miR-new_2-5p*; *sma-miR-new_4-5p*; *sma-miR-new_12-5p* and *sma-miR-new_13-3p*) seems to be directly linked to the oxidative phosphorylation process that occurs in the inner membrane of mitochondria. We suggest that the miRNAs *sma-miR-new_2-5p*; *sma-miR-new_4-5p*; *sma-miR-new_12-5p* and *sma-miR-new_13-3p* may play an important role in the post-transcriptional regulation of genes that are related to oxidative phosphorylation at the mitochondrial level.

In this context, our results revealed seven miRNA target genes that are involved in this biological process. Three of these predicted target genes are NADH ubiquinone oxidoreductase subunits that participate in the assembly and electron transport chain of the enzymatic complex I in mitochondria: *NADH dehydrogenase (ubiquinone) Fe-S protein 7*, *NADH dehydrogenase (ubiquinone) 1 beta* and *NADH:ubiquinone oxidoreductase complex I*. Other interesting miRNA target genes predicted were *cytochrome C type heme lyase*, *ATP synthase lipid-binding protein-like protein*, *ubiquinone biosynthesis protein* and *mitochondrial carrier 2 protein*. Cytochrome C type heme lyase is involved in the final maturation of cytochrome C1 (Complex IV)^25^ while ATP synthase lipid binding protein-like protein is linked to ATP synthesis, accordingly to gene ontology. Additionally, the *Ubiquinone biosynthesis protein* is directly related to the synthesis of ubiquinone (Coenzyme Q), which is a lipid that transports electrons in the respiratory chain.^26^ We suggest that the transcript levels of these genes under miRNA regulation might influence the transition of aerobic to anaerobic metabolism and vice-versa in the developmental stages of the parasite (Fig. 3).

Additionally, we performed qRT-PCR to validate the expression profiles of the target genes. We observed an up-regulation of *SmNADH dehydrogenase Fe-S protein 7*, *SmNADH dehydrogenase 1 beta subcomplex subunit 8*, *Smcytochrome c type heme lyase* and *SmATP synthase lipid-binding protein-like protein* in adult worms compared to other stages, suggesting that the miRNAs that target these genes may be potential regulators of their transcriptional levels mainly in larval stages. Once the miRNAs have different half-lives that affect their level of accumulation, differential stability and a variety of targets, we cannot exclude the hypothesis that they’re putative regulators of the targets predicted here. We could see a reverse tendency in the expression of *sma- miR-new_13-3p* and its putative target *NADH:ubiquinone oxidoreductase complex I*. The expression rates of *sma- miR-new_13-3p* were MTS 24 h > cercariae > MTS 3.5 h > adult worms, and the expression of *NADH:ubiquinone oxidoreductase complex I* gene was MTS 3.5 h > adult worms > cercariae > MTS 24 h.

Examining the *sma-miR-new_12-5p*; *sma-miR-new_13-3p* and *sma-miR-new_13-5p* miRNAs target genes, we also observed targets e related to other important biological processes in the parasite, including a *putative tetraspanin protein* (*TSP*), *cathepsin B 1 protein* (*CB1*), *venom allergen-like 6 protein* (*VAL-6*) and a *proteasome inhibitor subunit* (PI31). Tetraspanin proteins have been studied as protective antigens against schistosomiasis.^27^ The *S. mansoni* cathepsin B1 (*Sm*CB1) is a gut-associated peptidase that digests blood proteins as a source of nutrients for growth, development and reproduction in adult schistosomes. The inhibition of CB1 represents an attractive alternative for anti-schistosomal anti-drug development.^28^ VAL-6 was found in proteomic identification from the adult tegument as a member of a gene family called venom-allergen-like proteins that are present in platyhelminth species, which integrates the VAL group 2 (*Sm*VAL 6, 11, 13, 16 and 17) and has a potential role in modulating the evasion of the host immune system.^29^ PI31 plays an important role as a proteasome inhibitor in adult worms and is expressed during the life cycle of *S. mansoni*.^30^ The observations led us to suggest that there is a negative correlation between the expression profiles of *sma-miR-new_2-5p*; *sma-miR-new_13-3p*; *sma-miR-new_4-5p* and their putative targets genes. The fact that the miRNAs *sma-miR-new_12-5p*; *sma-miR-new_13-3p* and *sma-miR-new_13-5p* are down-regulated in adult worms might be due to the higher requirements of TSP, SmCB1 and SmVAL-6 transcript levels in these stages compared with schistosomula, once adult worms require an abundant amount of these proteins for their growth, development and immune system evasion. Although these target genes are expressed in schistosomula, the higher expression of *sma-miR-new_12-5p*; *sma-miR-new_13-3p* and *sma-miR-new_13-5p* in this stage might be related to the regulation of their abundance at post-transcriptional levels.

**Fig. 5:**
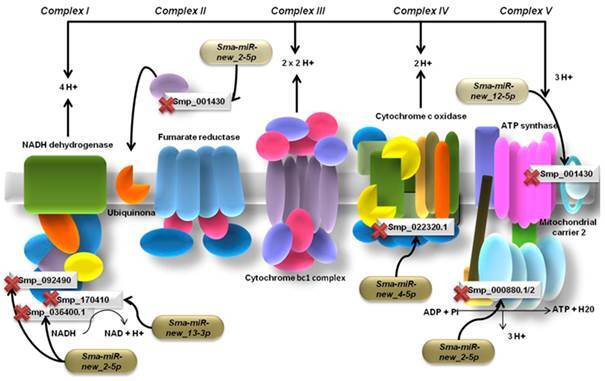
putative miRNAs that are involved in the regulation of oxidative phosphorylation in *Schistosoma mansoni*. miRNAs target enzymatic complexes and associated proteins in the electron transport chain that occurs in the inner membrane of mitochondria by transduction inhibition at post-transcriptional level. The targets entries are shown in Table.

Finally, we identified the TGF-beta signal transducer *smad2* as a putative target gene of the conserved *sma-miR-281*. One of the essential components of the TGF- β signaling pathway are the Smad proteins, which function as receptor substrates that translocate to the nucleus when activated and interact with a set of partner and regulatory proteins that will determine which genes are to be regulated.^31^ ^,^ ^32^ Here we suggest that *sma-miR-281* plays an important role in the post-transcriptional regulation of Smad2, primarily in male worms, once the deep sequencing analysis indicated that *sma-miR-281* has a sex-biased expression and is absent in females.^12^ The post-transcriptional regulation of TGF-β signaling components might directly reflect on the interactions between host-parasites and particularly by signal transduction from the male to female worms (Fig. 5).

*In conclusion* - In this work we evaluated for the first time the expression profiles of a set of miRNAs in *S. mansoni*. Our results contribute to the generation of more data and greater knowledge regarding the presence of miRNAs in parasite genomes. Among the miRNAs evaluated here, some showed no conservation with other organisms, and these results raise questions regarding whether these non-conserved miRNAs are in fact species-specific. Interestingly, most of the miRNA target genes that were predicted are related to important biological processes, such as TGF-β signaling, oxidative phosphorylation, immune system evasion, glucose and lipid metabolism, which suggests that mechanisms of gene regulation are important for driving the parasite development and the interactions between host-parasites). The differential expression of miRNAs and their putative targets, especially those ones related do oxidative phosphorylation, suggests a crucial role of post-transcriptional regulation in the energetic metabolism inversion, primarily in the transition of cercariae to schistosomula. Together, our results suggest that miRNAs might play important roles in post-transcriptional gene regulation in *S. mansoni* and might even act in synergism to regulate important metabolic pathways in the parasite.
