# Supplementary material for: Differential expression profiles of miRNAs and their putative targets in Schistosoma mansoni during its life cycle
Source: Mem Inst Oswaldo Cruz. 2021 May 14;116:e200326. doi: 10.1590/0074-02760200326 (PMC8128373; doi:10.1590/0074-02760200326)
Supplement: Supplementary file 1 [file 1678-8060-mioc-116-e200326-s.pdf]

TABLE I

miRNAs primer sequences

miRNA	pb	Strand	Primer sequence
sma-miR-281 MIMAT0033593	22	3	TGTCATGGAGTTGCTCTCTATA
sma-miR-250	22	3	CCTTCAGTTGACTCATGATCTC
sma-miR-2162-3p MIMAT0033666	21	3	TATTATGCAACGTTTCACTCT
sma-miR-92a	22	5	GATTGCACTAGTCACGGCTTTT
sma-miR-new_2-5p	22	5	TGGAAAACCTGTGAAAGTACTG
sma-miR-new_4-3p	22	3	TCGCTTTACCCATATCTGCTAG
sma-miR-new_4-5p	22	5	TGCAGGTAAAGTAATGCTTGTT
sma-miR-new_5-5p	22	5	TCCAAAGTTTCGTCCAGCAAAC
sma-miR-new_12-5p	22	5	ATCACAGCTCACACACAATTAA
sma-miR-new_13-3p	22	3	TTTTCTATGATGGTCTAGCTTC
sma-miR-new_13-5p	22	5	AGCTAGACTACCATGGAAAACT
sma-miR-new_16-3p	22	3	ATTACAAGCGATCACTTTTATA

TABLE II

Putative target accession numbers and primers used in quantitative reverse transcription-polymerase chain reaction (qRT-PCR)

Putative protein	Acession number	Primers
NADH dehydrogenase (ubiquinone) Fe-S protein 7	Smp_092490	F: 5'GCCAAATGACTCAACCGAAG3' R: 5'CGCAGATGGAG-GACAACC3'
NADH dehydrogenase (ubiquinone) 1 beta subcomplex subunit 8	Smp_036400	F: 5'CGCTACAATCTCTTGCCTTC3' R: 5'GATGGGTAGT-CATAGGGTTC3'
NADH:ubiquinone oxidoreductase complex I	Smp_170410	F: 5'TTGAAGGGCGATCTGTAG3' R: 5'CATCTGGTTGTGGGTTGAG3'
ubiquinone biosynthesis protein	Smp_001430	F: 5'TTGAAGGGCGATCTGTAG3' R: 5'CATCTGGTTGTGGGTTGAG3'
cytochrome c type heme lyase	Smp_022320.1	F: 5'ACCTTTCCCACTGTCTACTG3' R: 5'ACGCCAACCTTTACGAAG3'
ATP synthase lipid- binding protein-like protein	Smp_000880.1/2	F: 5'GGATCAGGTGCTGGTATTG3' R: 5'CCCATT-GCTTCACTCAGAG3'
ubiquinone biosynthesis protein	Smp_001430	F: 5'CCTACTGACATGCTTGGTG3' R: 5'CGCAGAGAACCAAAGTAACC3'

CC BY

TABLE III

Adult				
Gene	Triplicate	MeanCqc	Δ Cq	2^-Δ Cq
U6	14.6448	14.4723	0.1725	0.887304
U6	14.2998	14.4723	-0.1725	1.12701
U6	14.4723	14.4723	0	1
mir-2162	19.6965	14.4723	5.2242	0.026752
mir-2162	19.3038	14.4723	4.8315	0.035122
mir-2162	19.5001	14.4723	5.0278	0.030654
mir-250	23.4225	14.4723	8.9502	0.002022
mir-250	22.3251	14.4723	7.8528	0.004326
mir-250	22.8738	14.4723	8.4015	0.002957
mir-281	20.6947	14.4723	6.2224	0.013393
mir-281	19.6331	14.4723	5.1608	0.027954
mir-281	20.1639	14.4723	5.6916	0.019349
mir-92a	27.4608	14.4723	12.9885	0.000123
mir-92a	27.0643	14.4723	12.592	0.000162
mir-92a	27.2625	14.4723	12.7902	0.000141
mir-2_5p	26.9482	14.4723	12.4759	0.000176
mir-2_5p	26.9227	14.4723	12.4504	0.000179
mir-2_5p	26.9354	14.4723	12.4631	0.000177
mir-4_3p	21.362	14.4723	6.8897	0.008433
mir-4_3p	19.8496	14.4723	5.3773	0.024059
mir-4_3p	20.6058	14.4723	6.1335	0.014244
mir-4_5p	22.2173	14.4723	7.745	0.004661
mir-4_5p	22.0479	14.4723	7.5756	0.005242
mir-4_5p	22.1326	14.4723	7.6603	0.004943
mir-5_5p	24.7681	14.4723	10.2958	0.000796
mir-5_5p	23.4758	14.4723	9.0035	0.001948
mir-5_5p	24.1219	14.4723	9.6496	0.001245
mir-12_5p	27.8647	14.4723	13.3924	9.3E-05
mir-12_5p	26.3436	14.4723	11.8713	0.000267
mir-12_5p	27.1041	14.4723	12.6318	0.000158
mir-13_3p	19.7472	14.4723	5.2749	0.025828
mir-13_3p	20.3475	14.4723	5.8752	0.017037
mir-13_3p	20.0473	14.4723	5.575	0.020978
mir-13_5p	22.1389	14.4723	7.6666	0.004922
mir-13_5p	21.7038	14.4723	7.2315	0.006654
mir-13_5p	21.9213	14.4723	7.449	0.005723
mir-16_3p	34.0559	14.4723	19.5836	1.27E-06
mir-16_3p	33.9251	14.4723	19.4528	1.39E-06
mir-16_3p	33.9905	14.4723	19.5182	1.33E-06

Egg				
Gene	Triplicate	MeanCqc	Δ Cq	2^-Δ Cq
U6	15.5956	15.5282	0.0674	0.954356
U6	15.4608	15.5282	-0.0674	1.047827
U6	15.5282	15.5282	0	1
mir-2162	23.2668	15.5282	7.7386	0.004682
mir-2162	22.3536	15.5282	6.8254	0.008818
mir-2162	22.8102	15.5282	7.282	0.006425
mir-250	25.2476	15.5282	9.7194	0.001186
mir-250	24.015	15.5282	8.4868	0.002788
mir-250	24.6313	15.5282	9.1031	0.001818
mir-281	26.2852	15.5282	10.757	0.000578
mir-281	25.1406	15.5282	9.6124	0.001278
mir-281	25.7129	15.5282	10.1847	0.000859
mir-92a	30.7892	15.5282	15.261	2.55E-05
mir-92a	29.2743	15.5282	13.7461	7.28E-05
mir-92a	30.0337	15.5282	14.5055	4.3E-05
mir-2_5p	29.0739	15.5282	13.5457	8.36E-05
mir-2_5p	29.7143	15.5282	14.1861	5.36E-05
mir-2_5p	29.3941	15.5282	13.8659	6.7E-05
mir-4_3p	23.9517	15.5282	8.4235	0.002913
mir-4_3p	22.6689	15.5282	7.1407	0.007087
mir-4_3p	23.3103	15.5282	7.7821	0.004543
mir-4_5p	26.1359	15.5282	10.6077	0.000641
mir-4_5p	26.4496	15.5282	10.9214	0.000516
mir-4_5p	26.2927	15.5282	10.7645	0.000575
mir-5_5p	27.0119	15.5282	11.4837	0.000349
mir-5_5p	26.0523	15.5282	10.5241	0.000679
mir-5_5p	26.5321	15.5282	11.0039	0.000487
mir-12_5p	27.224	15.5282	11.6958	0.000301
mir-12_5p	28.3745	15.5282	12.8463	0.000136
mir-12_5p	27.7992	15.5282	12.271	0.000202
mir-13_3p	23.9455	15.5282	8.4173	0.002925
mir-13_3p	24.2721	15.5282	8.7439	0.002333
mir-13_3p	24.1088	15.5282	8.5806	0.002612
mir-13_5p	23.9455	15.5282	8.4173	0.002925
mir-13_5p	24.2721	15.5282	8.7439	0.002333
mir-13_5p	24.1088	15.5282	8.5806	0.002612
mir-16_3p	32.4827	15.5282	16.9545	7.87E-06
mir-16_3p	33.0995	15.5282	17.5713	5.13E-06
mir-16_3p	32.7911	15.5282	17.2629	6.36E-06

Cercariae				
Gene	Triplicate C	MeanCqc	Δ Cq	2^-Δ Cq
U6	16.5648	16.2196	0.3452	0.787199
U6	15.8744	16.2196	-0.3452	1.270327
U6	16.2196	16.2196	0	1
mir-2162	25.0289	16.2196	8.8093	0.002229
mir-2162	21.391	16.2196	5.1714	0.027749
mir-2162	23.2099	16.2196	6.9903	0.007865
mir-250	28.4233	16.2196	12.2037	0.000212
mir-250	26.4492	16.2196	10.2296	0.000833
mir-250	27.4362	16.2196	11.2166	0.00042
mir-281	24.4407	16.2196	8.2211	0.003351
mir-281	25.4949	16.2196	9.2753	0.001614
mir-281	24.9678	16.2196	8.7482	0.002326
mir-92a	32.7206	16.2196	16.501	1.08E-05
mir-92a	33.9179	16.2196	17.6983	4.7E-06
mir-92a	33.3192	16.2196	17.0996	7.12E-06
mir-2_5p	28.4489	16.2196	12.2293	0.000208
mir-2_5p	28.692	16.2196	12.4724	0.000176
mir-2_5p	28.5704	16.2196	12.3508	0.000191
mir-4_3p	26.8436	16.2196	10.624	0.000634
mir-4_3p	24.1543	16.2196	7.9347	0.004087
mir-4_3p	25.4989	16.2196	9.2793	0.001609
mir-4_5p	30.1412	16.2196	13.9216	6.44E-05
mir-4_5p	27.4634	16.2196	11.2438	0.000412
mir-4_5p	28.8023	16.2196	12.5827	0.000163
mir-5_5p	25.8873	16.2196	9.6677	0.00123
mir-5_5p	26.68	16.2196	10.4604	0.00071
mir-5_5p	26.2836	16.2196	10.064	0.000934
mir-6_3p	30.2871	16.2196	14.0675	5.82E-05
mir-12_5p	28.3394	16.2196	12.1198	0.000225
mir-12_5p	27.7397	16.2196	11.5201	0.00034
mir-12_5p	28.0395	16.2196	11.8199	0.000277
mir-13_3p	20.0172	16.2196	3.7976	0.071913
mir-13_3p	19.4044	16.2196	3.1848	0.109971
mir-13_3p	19.7108	16.2196	3.4912	0.088929
mir-13_5p	23.0289	16.2196	6.8093	0.008917
mir-13_5p	23.0557	16.2196	6.8361	0.008752
mir-13_5p	23.2403	16.2196	7.0207	0.007701
mir-16_3p	30.9611	16.2196	14.7415	3.65E-05
mir-16_3p	30.4094	16.2196	14.1898	5.35E-05
mir-16_3p	30.6852	16.2196	14.4656	4.42E-05

Shistosomula 3,5 h				
Gene	Triplicate C	MeanCqc	Δ Cq	2^-Δ Cq
U6	17.6062	17.7135	-0.1073	1.07721
U6	17.8208	17.7135	0.1073	0.928324
U6	17.7135	17.7135	0	1
mir-2162	22.1831	17.7135	4.4696	0.045135
mir-2162	21.4588	17.7135	3.7453	0.074568
mir-2162	21.8209	17.7135	4.1074	0.058016
mir-250	28.1827	17.7135	10.4692	0.000705
mir-250	25.1775	17.7135	7.464	0.005664
mir-250	26.6801	17.7135	8.9666	0.001999
mir-281	25.0732	17.7135	7.3597	0.006088
mir-281	24.3499	17.7135	6.6364	0.010052
mir-281	24.7115	17.7135	6.998	0.007823
mir-92a	33.3271	17.7135	15.6136	1.99E-05
mir-92a	33.3894	17.7135	15.6759	1.91E-05
mir-92a	33.2582	17.7135	15.5447	2.09E-05
mir-2_5p	27.56	17.7135	9.8465	0.001086
mir-2_5p	27.5271	17.7135	9.8136	0.001111
mir-2_5p	27.5435	17.7135	9.83	0.001099
mir-4_3p	26.4017	17.7135	8.6882	0.002424
mir-4_3p	24.809	17.7135	7.0955	0.007312
mir-4_3p	25.6053	17.7135	7.8918	0.00421
mir-4_5p	30.7844	17.7135	13.0709	0.000116
mir-4_5p	30.7844	17.7135	13.0709	0.000116
mir-4_5p	30.7844	17.7135	13.0709	0.000116
mir-5_5p	29.0595	17.7135	11.346	0.000384
mir-5_5p	27.3787	17.7135	9.6652	0.001232
mir-5_5p	28.2191	17.7135	10.5056	0.000688
mir-12_5p	28.3706	17.7135	10.6571	0.000619
mir-12_5p	25.9178	17.7135	8.2043	0.00339
mir-12_5p	27.1442	17.7135	9.4307	0.001449
mir-13_3p	21.3715	17.7135	3.658	0.07922
mir-13_3p	21.3483	17.7135	3.6348	0.080504
mir-13_3p	21.3599	17.7135	3.6464	0.079859
mir-13_5p	23.262	17.7135	5.5485	0.021367
mir-13_5p	23.3781	17.7135	5.6646	0.019714
mir-13_5p	23.32	17.7135	5.6065	0.020525
mir-16_3p	32.0011	17.7135	14.2876	5E-05
mir-16_3p	31.2129	17.7135	13.4994	8.64E-05
mir-16_3p	31.607	17.7135	13.8935	6.57E-05

Schistosomula 24 h				
Gene	Triplicate	MeanCqc	Δ Cq	2^-Δ Cq
U6	18.5869	19.1342	-0.5473	1.461348
U6	19.6815	19.1342	0.5473	0.6843
U6	19.1342	19.1342	0	1
mir-2162	25.1159	19.1342	5.9817	0.015824
mir-2162	22.4041	19.1342	3.2699	0.103672
mir-2162	23.76	19.1342	4.6258	0.040504
mir-250	29.1552	19.1342	10.021	0.000962
mir-250	28.4492	19.1342	9.315	0.00157
mir-250	28.8022	19.1342	9.668	0.001229
mir-281	24.2319	19.1342	5.0977	0.029204
mir-281	24.7674	19.1342	5.6332	0.020148
mir-281	24.4996	19.1342	5.3654	0.024258
mir-92a	32.3405	19.1342	13.2063	0.000106
mir-92a	27.008	19.1342	7.8738	0.004263
mir-92a	29.6742	19.1342	10.54	0.000672
mir-2_5p	31.6237	19.1342	12.4895	0.000174
mir-2_5p	30.0417	19.1342	10.9075	0.000521
mir-2_5p	30.8327	19.1342	11.6985	0.000301
mir-4_3p	28.8882	19.1342	9.754	0.001158
mir-4_3p	29.1362	19.1342	10.002	0.000975
mir-4_3p	29.0122	19.1342	9.878	0.001063
mir-4_5p	29.0761	19.1342	9.9419	0.001017
mir-4_5p	28.7304	19.1342	9.5962	0.001292
mir-4_5p	28.9032	19.1342	9.769	0.001146
mir-5_5p	30.5699	19.1342	11.4357	0.000361
mir-5_5p	31.1227	19.1342	11.9885	0.000246
mir-5_5p	30.8463	19.1342	11.7121	0.000298
mir-6_3p	33.4381	19.1342	14.3039	4.94E-05
mir-6_3p	31.3687	19.1342	12.2345	0.000208
mir-6_3p	32.4034	19.1342	13.2692	0.000101
mir-12_5p	29.6688	19.1342	10.5346	0.000674
mir-12_5p	29.0516	19.1342	9.9174	0.001034
mir-12_5p	29.3602	19.1342	10.226	0.000835
mir-13_3p	22.094	19.1342	2.9598	0.128532
mir-13_3p	22.8186	19.1342	3.6844	0.077783
mir-13_3p	22.4563	19.1342	3.3221	0.099988
mir-13_5p	23.8675	19.1342	4.7333	0.037595
mir-13_5p	24.1202	19.1342	4.986	0.031555
mir-13_5p	23.9938	19.1342	4.8596	0.034444
mir-16_3p	34.0759	19.1342	14.9417	3.18E-05
mir-16_3p	37.6093	19.1342	18.4751	2.74E-06
mir-16_3p	35.8426	19.1342	16.7084	9.34E-06
